# Multicolor Photodetector of a Single Er^3+^-Doped CdS Nanoribbon

**DOI:** 10.1186/s11671-015-0975-3

**Published:** 2015-07-08

**Authors:** Hou Dedong, Liu Ying-Kai, De-Peng Yu

**Affiliations:** Institute of Physics and Electronic Information, Yunnan Normal University, No. 768 Juxian Street, Chenggong New District, Kunming, 650500 People’s Republic of China; Key Laboratory of Yunnan Higher Education Institutes for Optoelectric Information & Technology, Kunming, 650500 People’s Republic of China; Key Laboratory of Yunnan Normal University for Photoelectric Materials & Device, Kunming, 650500 People’s Republic of China; State Key Laboratory for Mesoscopic Physics, Department of Physics, Peking University, No. 5 Yiheyuan Road, Haidian District, Beijing, 100871 People’s Republic of China

**Keywords:** Multicolor photodetector, Er^3+^-doped CdS nanoribbons, Near-infrared light

## Abstract

Er^3+^-doped CdS nanoribbons (Er-CdS NRs) are synthesized by thermal evaporation and then characterized by field emission scanning electron microscopy (FE-SEM), high-resolution transmission electron microscopy (HRTEM), photoluminescence (PL), and absorption spectra. The Er-CdS NR photodetector is studied systematically, including spectral response, light intensity response, and photoconductance (*G*) versus temperature (*T*). It is found that Er-CdS NR has the ability of detecting multicolor light including blue, red, and near-infrared light with higher responsivity (*R*_*λ*_) and external quantum efficiency (*η*). The conductance of Er-CdS NR under dark conditions decreases with increasing temperature in the range of 87–237 K, while its conductance increases with increasing temperature in the range of 237–297 K when *T* is larger than 237 K. These results indicated that ionized impurities and the intrinsic excitation are responsible for the conductance change of Er-CdS NR in the dark. The superior performance of the Er-CdS NR device offers an avenue to develop highly sensitive multicolor photodetector applications.

**Electronic supplementary material:**

The online version of this article (doi:10.1186/s11671-015-0975-3) contains supplementary material, which is available to authorized users.

## Background

Semiconductor nanostructures, such as nanowires [1], nanobelts [2], colloidal quantum dots [3], and polymer-inorganic nanocrystal composites [4], are attractive building blocks for a new generation of high-sensitivity and high-selectivity sensors primarily because of their high surface-to-volume ratios and diverse functions as both device elements and interconnects [5, 6]. As an important application of semiconductor materials, photodetectors, optical switches, or nanoelectromechanical (NEM) switches are essential elements in imaging techniques and light wave communications and possibly in future memory storage as well as optoelectronic circuits [1–7]. Among them, some semiconductors such as ZnS [2] and ZnO [8] have been assembled into nanometer “visible-light-blind” or “solar-blind” ultraviolet (UV) light sensors with high sensitivity and selectivity. Some photodetectors have achieved a broad spectral response, such as monolayer MoS_2_, in which the photocurrent monotonously increased as the wavelength of the incident light decreased from 680 to 400 nm [9]. Generally, every material only detects UV light (GaN) [10], green light (CdS) [11], or near-infrared light (CdSe) [12]. Furthermore, slow modulation response speed and narrow response bandwidth (<20 Hz) have been tolerated in these devices, but these limitations severely curtail potential applications. Pan et al. reported that a high-performance photodetector based on the CdS_0.49_Se_0.51_/CdS_0.91_Se_0.09_ lateral heterostructure has been designed to detect double spectral response bands, with the peaks at around 525 and 602 nm, respectively [13]. In fact, we hope that photodetectors can detect not only UV light but also near-infrared light, even tricolor light. Recently, a far-infrared photodetector in a silicon-doped vanadium material has been investigated, and it was found that as the V concentration increases, an important increase of the photoresponse is observed in the far-infrared region of the spectrum [14]. This idea naturally reminds us of doping.

Doping, the intentional incorporation of impurities into materials, is a primary means of tuning electronic, optical, and magnetic properties of bulk semiconductors. As you know, rare earth (RE) elements are effective luminescent centers for RE-doped semiconductors because the excitation of RE ions can occur by the recombination of the photogenerated carriers confined in semiconductors and subsequent energy transfer to RE ions [15]. Therefore, RE-doped II–VI materials are promising candidates for application in color thin-film electroluminescence devices, nonlinear optics, and multicolor optical switches [16, 17]. Rare earth doping may play an important role in obtaining highly efficient multicolor photodetectors and upconversion signals.

Based on these reasons, we have synthesized Er^3+^-doped CdS nanoribbons (hereafter referred to as Er-CdS NRs) via thermal evaporation and then investigate photoconductance (PC) of a single Er-CdS NR device. It is found that a single Er-CdS NR device detects not only blue and red light but also the infrared one. Its photoconductance could be tuned over 4, 3, and 2 orders of magnitude illuminated by blue, red, and near-infrared light, respectively. Er-CdS NRs offer a promising platform for multicolor photodetectors with high rate and efficiency of detection due to the fine regulation of their band structure coming from numerous valence states of RE ions. In addition, we study the PC dependence of the single Er-CdS NR on temperature to reveal its photodetection mechanism.

## Methods

### Preparation of Er-CdS Nanoribbons

CdS nanoribbons were synthesized in a horizontal tube furnace with three temperature zones via thermal evaporation. The premixed powders of CdS powers (Aldrich, purity 99.99 %) and erbium(III) acetate hydrate (Aldrich, purity 99.9 %) were placed at the center of an alumina tube. Au-coated silicon substrates were placed at the downstream position of the source material. After that, the tube was evacuated to a base pressure of 5 × 10^−6^ Torr and then the sources were heated to 840 °C at a rate of 40 °C/min; this temperature was maintained for 2.5 h. A carrier gas of high-purity argon premixed with 5 % hydrogen was fed at a total flow rate of 20 sccm. The pressure inside the alumina tube was maintained at 150 Torr during the whole experimental process. The as-synthesized nanoribbons were bright yellow in color.

### Characterization

These nanoribbons were characterized by scanning electron microscopy (SEM, Quanta 250 FEG) and high-resolution transmission electron microscopy (HRTEM, CM200 FEG operating at 200 kV). Room-temperature photoluminescence (PL) was measured by using the fourth harmonic of a Nd:YAG laser with a wavelength of 244 nm for excitation and a 0.5-m monochromator with an expected spectral resolution of 0.1 nm. The absorption spectra of Er-CdS NRs were measured using a spectrometer (PerkinElmer, Lambda 2S) by dispersing the nanoribbons in alcohol.

### Fabrication and Characterization of a Single Er-CdS NR Device

For the fabrication of a single nanoribbon detector, Er-CdS NRs were dispersed into dehydrated ethyl acetate by ultrasonic processes. Subsequently, the suspension solution was dropped on a p-type Si substrate with a SiO_2_ (500 nm) layer on top, and then a desired NR density was obtained on this substrate. After drying the wafer and locating the position of Er-CdS NRs, patterned Ti (20 nm) and Au (100 nm) electrodes were successively deposited on the two ends of the nanoribbons in high vacuum by e-beam evaporation with the assistance of a mesh-grid mask composed of tungsten wires (10 μm in diameter). Since the lengths of the nanoribbons were larger than the diameter of tungsten wires, the electrodes were formed on the uncovered parts of nanoribbons. Thus, a single Er-CdS NR device was obtained.

### Photoconductance Measurements

A light system combining a mercury lamp (500 W) and a monochromator (1/4 m, VIS-NIR Cornerstone 260) was used to provide the monochromatic light, which was focused and guided onto the nanoribbons perpendicularly. Current-voltage (*I*-*V*) measurements were performed by using a two-probe configuration. The dependence of the conductance on the temperature was measured at a temperature of 87–297 K. To measure the response time of the nanoribbon photodetector to light irradiation, a mechanical chopper (frequency ranging from 1 to 500 Hz) was used to turn on and off the light irradiation.

## Results and Discussion

**Fig. 1:**
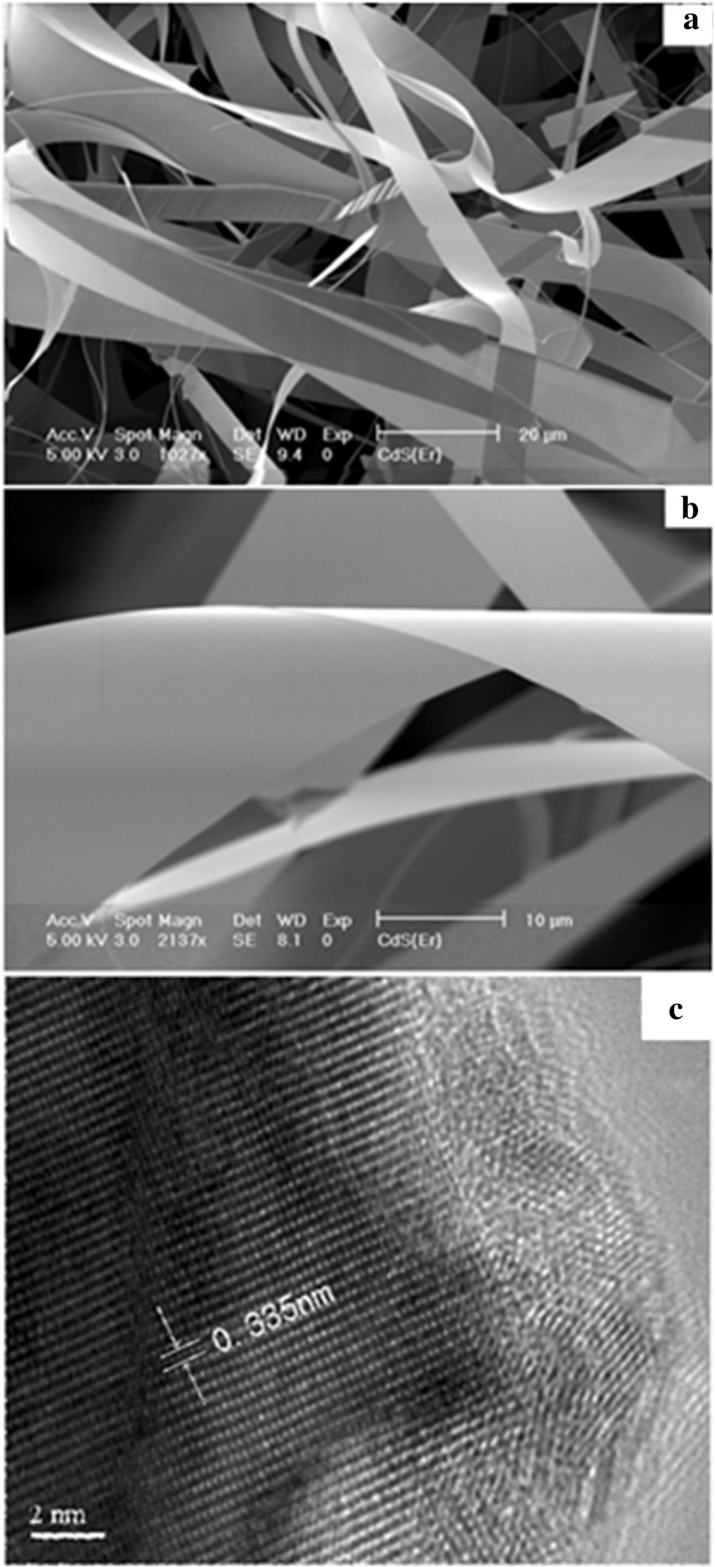
SEM and HRTEM images of the as-synthesized Er-CdS NRs. **a** SEM image of Er-CdS NRs at low magnification. **b** SEM image of a single Er-CdS NR at high magnification. **c** HRTEM image of a single Er-CdS NR

SEM morphology in Fig. 1a shows that the surfaces of CdS(Er) nanoribbons are clean and smooth. The width and thickness of the nanoribbons are in the range of 3–20 μm and 30–80 nm, respectively. The typical length of the ribbons is about 100–200 μm, and the length of some ribbons is up to 1 mm. But each ribbon is uniform in width and thickness along its length direction, as shown in Fig. 1b. The HRTEM image (Fig. 1c) indicates that the CdS nanoribbons are hexagonal single crystals growing along the [0002] orientation.

**Fig. 2:**
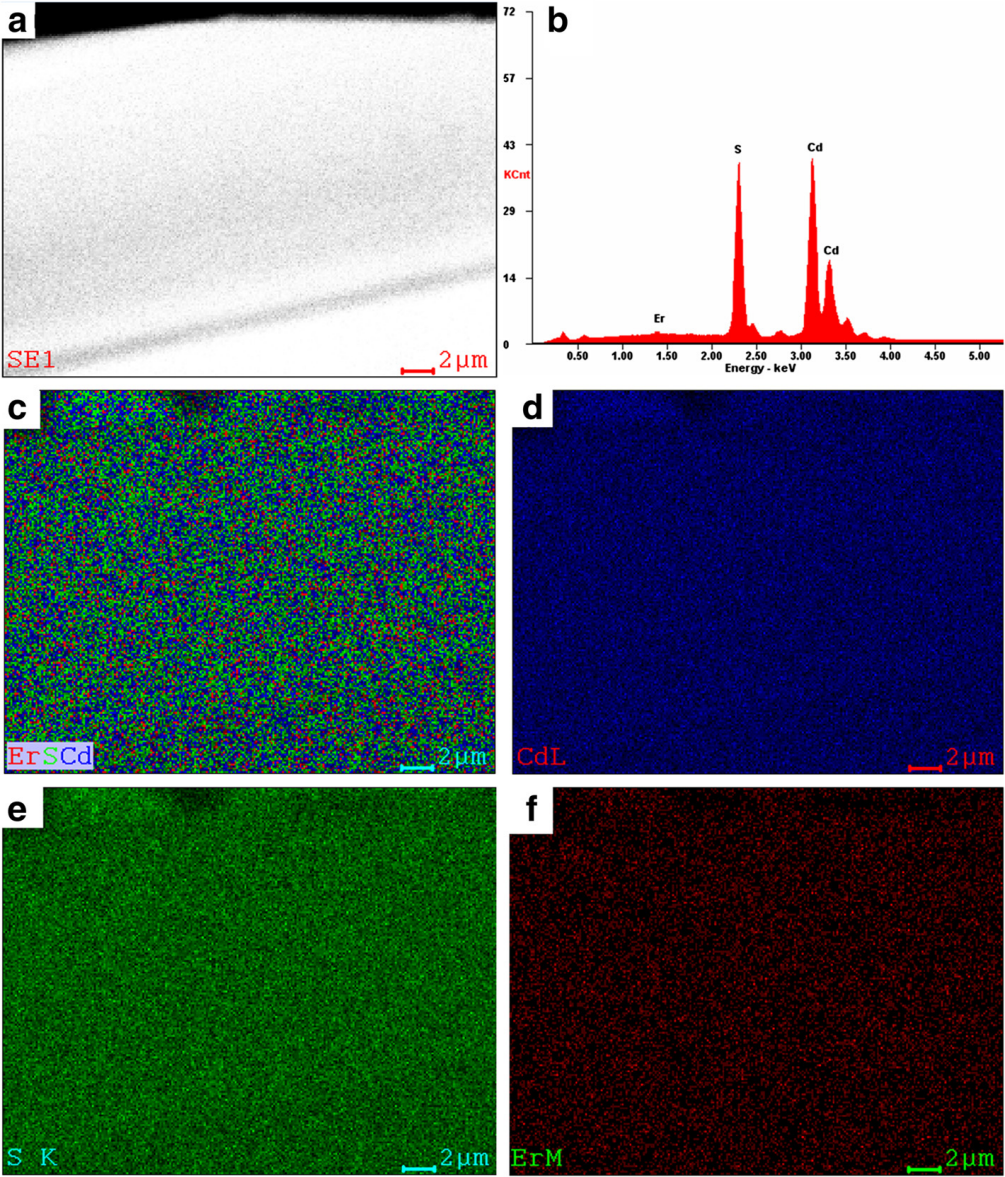
SEM, EDX, and elemental mappings. **a** SEM image of a single Er-CdS NR. **b** Corresponding SEM-EDX spectrum recorded from the nanoribbon. **c**–**f** 2D elemental mappings for the three detected elements (Er-CdS, Cd, S, and Er)

SEM combined with energy-dispersive X-ray spectroscopy (EDX) was used to investigate the microstructure and the spatial elemental composition of the obtained NRs. Figure 2a is a typical SEM image of a representative NR with a uniform width of ~18.5 μm. Figure 2b shows the EDX spectrum collected from the same nanoribbon (Fig. 2a), which reveals that the nanoribbon is considerably composed of Cd, S, and Er. The analysis gives the amounts of Cd, S, and Er which are 48.13, 50.32, and 1.55 at.%, respectively. Figure 2c–f shows two-dimensional (2D) elemental mappings of a selected region of this nanoribbon for the detected elements Cd, S, and Er, respectively. As can be seen, Cd, S, and Er are very homogeneously distributed across the whole nanoribbon.

**Fig. 3:**
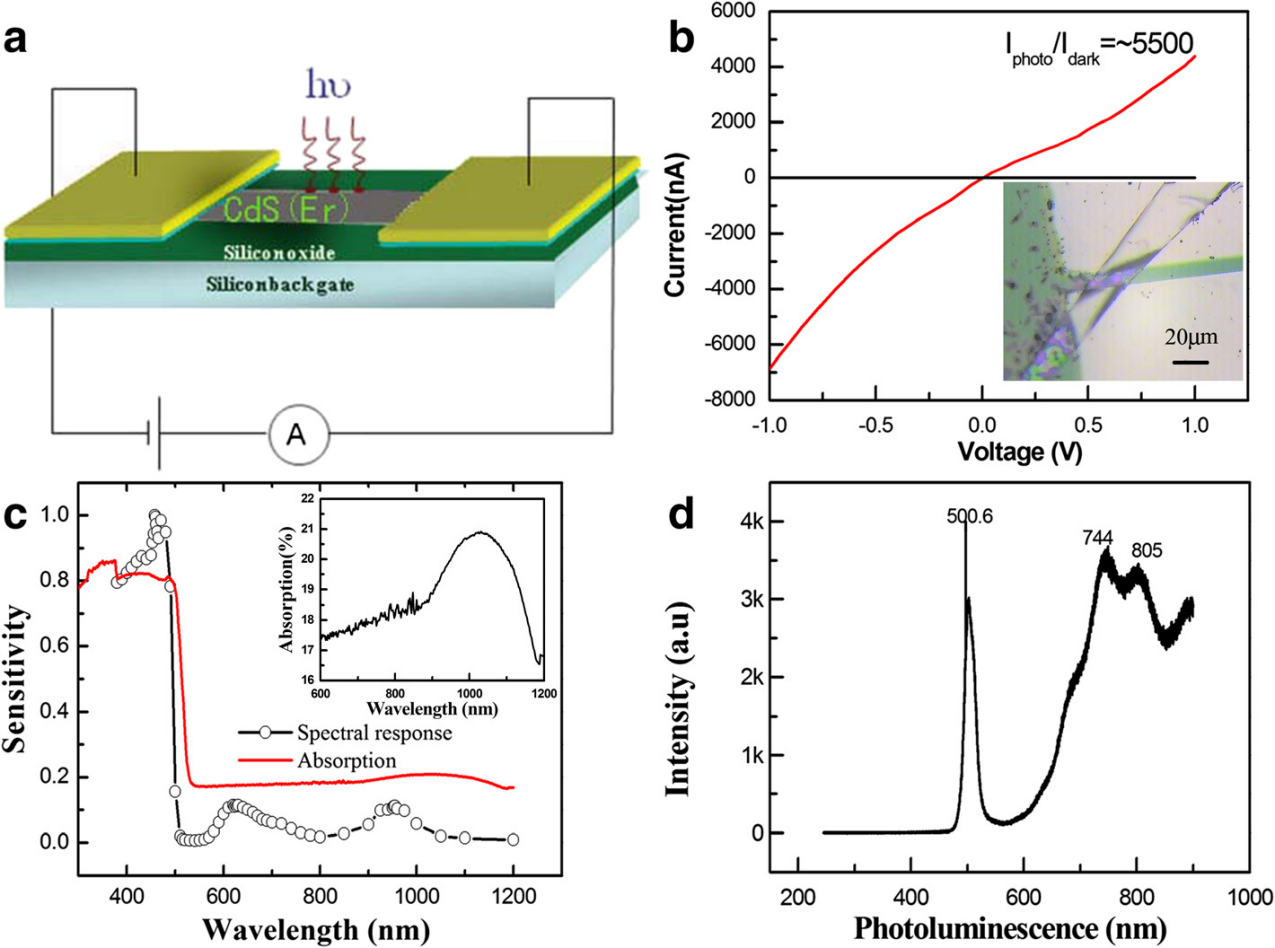
The detector’s photoresponse properties. **a** A schematic diagram of the detector configuration. **b** *I*-*V* curves of the Er-CdS NR device under illumination with an incandescent lamp; the inset shows the optical microscopic image of a single Er-CdS NR detector. **c** The spectra response and absorption spectra of the Er-CdS NR detector; the inset shows the absorption spectrum of the Er-CdS NR detector in the range of 600–1200 nm. **d** PL spectra of Er-CdS NRs

Figure 3a is a schematic diagram of the device configuration for photocurrent measurement, in which a monochromatic light (full width at half maximum (FWHM) 3 nm) is illuminated on the surface of the Er-CdS NR in the normal direction, and the *I*-*V* measurements are performed by using a two-probe method. In our experiments, Ti/Au parallel electrodes 10 μm apart are deposited on the nanoribbon dispersed on a p-type Si substrate with a 500-nm-thick SiO_2_ layer, and the uncovered part of the nanoribbon is exposed to the incident light. The inset of Fig. 3b displays the optical image of a fabricated Er-CdS NR detector with a width of 20.8 μm and a thickness of less than 70 nm. Its typical *I*-*V* curves under dark conditions and incandescent light illumination with a power density of 1.5 mW/cm^2^ are shown in Fig. 3b. It is seen that the photocurrent drastically increases under incandescent light illumination compared to the dark current. The approximate linear shape of the *I*-*V* curves suggests good ohmic contacts between the Er-CdS NR and Ti/Au electrodes. The PC ratio of the Er-CdS NR illuminated by incandescent light to that under dark conditions is 5500.

In order to obtain the detailed wavelength-related spectral response, we also measured the photocurrents of the device with the incident light wavelength scanning from 300 to 1350 nm. Figure 3c shows the wavelength-dependent photocurrent response of the device constructed with Er-CdS NR (working voltage 1 V, light density 25 μW/cm^2^). It can be seen that the Er-CdS NR device exhibits three spectral response bands, with the peaks at around 457.5, 620, and 955 nm. The spectral response peak at 457.5 nm is sharper than those at 620 and 955 nm, with the spectral FWHM of about 22.5, 36.5, and 51.4 nm. At the same time, the photocurrent under illumination of 457.5 nm light is much higher than those under illumination with light of 620 and 955 nm, respectively. As reported in the literatures, the infrared emission spectrum in Cs_3_Y_2_Br_9_ (1 % Er) is at 1548 nm related to the ^4^I_13/2_ → ^4^I_15/2_ [18, 19] and the strong absorption peak in Y_3_Sc_2_Ga_3_O_12_ (Er) [20] is at 1524.9 nm related to the ^4^I_15/2_ → ^4^I_13/2_, which match with IR detection behavior. Therefore, it is speculated that there is a fourth maximum photocurrent peak at the wavelength of 1540 nm. This result has not been proved due to the limit of the monochromator (the longest wavelength is 1350 nm). Therefore, a multicolor photodetector is obtained.

To clarify the origin of the spectral response, the absorption spectrum (red line) of Er-CdS NRs is measured, as depicted in Fig. 3c. It is seen that there is a broad absorption peak at 390–445 nm and a small sharp peak at around 500 nm. The former is related to the transformation of the ^4^I_15/2_ → ^4^F_3/2_ (^4^F_5/2_), and its absorption edge matches with the spectral response band at 457.5 nm. The latter corresponds to the energy band gap. We have carefully observed it and found that there is a steadily increasing absorption in the range of 500–800 nm, which is related to the transition of Er^3+^ ions with ^4^I_15/2_ → ^4^F_9/2_ [18, 19], but no obvious absorption peak is observed at 620 nm. However, there is a broad absorption peak at around 900–1100 nm, as shown in the inset of Fig. 3c, which is related to ^4^I_15/2_ → ^4^I_11/2_ transitions of Er^3+^ ions [18, 19]. Fascinatingly, the best response wavelengths are nearly coincident with the absorption spectrum at the absorption edge and at the long wavelength position, revealing that the response spectrum is directly related to the energy band structure of Er-CdS NRs, and the transitions of Er^3+^ ions energy levels, which is reflected by the photocurrent measurement. Thus, it can be concluded that the enhancements of the photoconductive response are due to the electron-hole pairs excited by the incident light with energy larger than the band gap of CdS and the transition energy of Er^3+^ ion energy levels. Light with a smaller energy has not enough energy to excite electrons from the valence band to the conduction band and thus contributes little to the photocurrent.

For comparisons, the photoluminescence spectrum for Er-CdS NRs is measured, which is displayed in Fig. 3d. It is seen that three emission bands are observed in green, red, and infrared regions at 504.5, 701, 742, 751, 806, and 894 nm. They were associated with ^4^F_9/2_ → ^4^I_15/2_, ^4^F_7/2_ → ^4^I_13/2_, ^4^I_9/2_ → ^4^I_15/2_, and ^4^I_11/2_ → ^4^I_15/2_ transitions of erbium ions [18–24]. As a consequence, incorporation of the dopant erbium ions into the matrix, rather than simple adhesion to the surface of the CdS nanoribbons, was demonstrated.

**Fig. 4:**
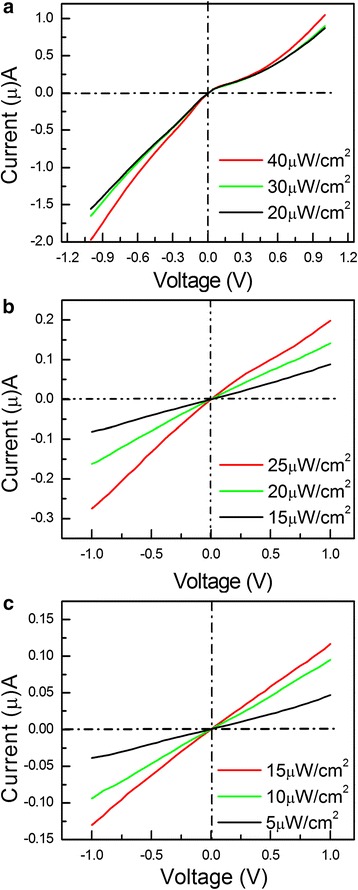
*I*-*V* curves of the Er-CdS NR detector under illuminations with different wavelength light. **a** 457.5 nm, **b** 620 nm, and (**c**) 955 nm

Figure 4 shows the *I*-*V* curves of the Er-CdS NR under irradiations of 457.5, 620, and 955 nm light with different power densities. The photocurrent increases with an increasing power intensity, but they exhibit a nonlinearity increase with an applied voltage in Fig. 4a. The kink point is at zero. However, the photocurrents change nearly linearly with the applied voltage in Fig. 4b upon 620 nm light illumination. The *I*-*V* curves do exhibit linear shape under illumination with light of 955 nm, as presented in Fig. 4c. These results imply that irradiations of different wavelength light can change the contact between the semiconductor nanoribbon and the metal electrodes.

**Fig. 5:**
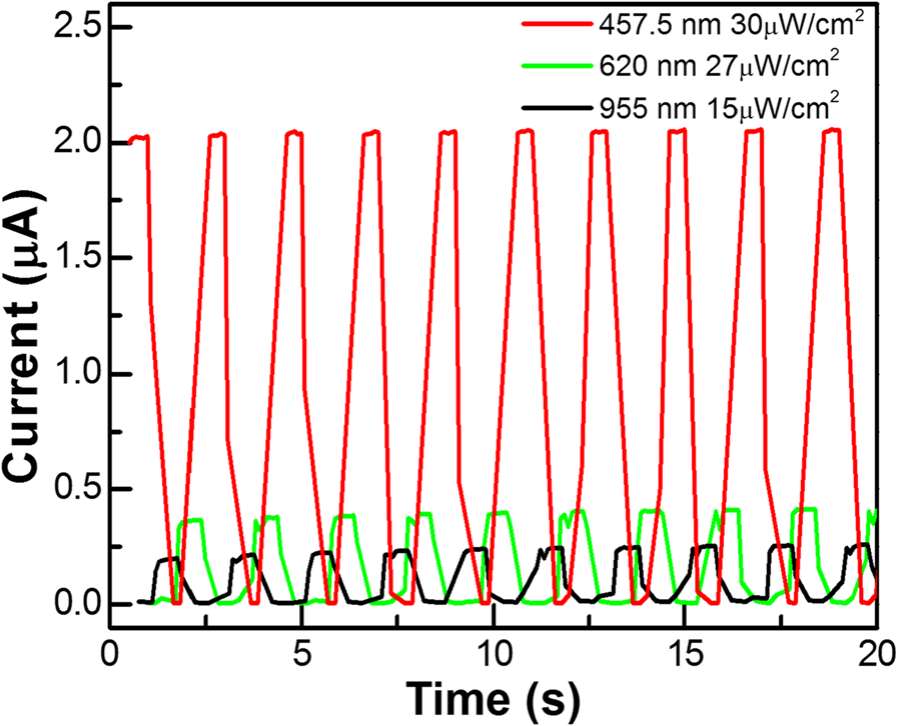
The time-dependent photoresponse of the Er-CdS NR detector

Figure 5 shows the time-dependent photoresponse of the Er-CdS NR detector, which is measured by periodically turning on and off the 457.5, 620, and 955 nm light at a bias voltage of 1 V. The results indicate that the device not only has high *I*_on_/*I*_off_ ratios as large as 10^3^, 10^2^, and 10 at the wavelengths of 457.5, 620, and 955 nm, corresponding to power densities of 30, 27, and 15 μW/cm^2^, respectively, but also exhibits a good reversible stability on switching properties. Figure 5 shows that the time-dependent photoresponses are similar under illumination with light of different wavelengths.

The spectral responsivity (*R*_*λ*_) and external quantum efficiency (EQE) (*η*) are critical parameters for high-performance photodetector applications. Large values of *R*_*λ*_ and *η* represent a high sensitivity of the device under specific light illumination. According to the definition of responsivity for a photodetector, *R*_*λ*_ = Δ*I*_ph_/(*P*_*λ*_*S*) and EQE = *hcR*_*λ*_/(*eλ*), where Δ*I*_ph_ is the current difference between the *I*_photo_ and the *I*_dark_ at a specific monochromatic light illumination, *P*_*λ*_ is the intensity of monochromatic light, *S* is the effective illuminated area, *h* is the Planck constant, *c* is the velocity of light, *e* is the electronic charge, and *λ* is the incident light wavelength. We calculated the corresponding *R*_*λ*_ and EQE values at the peak wavelength of our device. It is found that the *R*_*λ*_ of the Er-CdS NR device from this study are much higher than those of other nanostructure photodetectors such as InAs, InAs_*x*_P_1 *− x*_ [13], ZnS nanobelt (NB) [2], ZnSe nanowire (NW) [25], Sb_2_Se_3_ NW [26], Zn_2_GeO_4_ NW [27], and In_2_Se_3_ NW (α-phase/κ-phase) [28]. Its EQE are higher than those of some nanowire or nanobelt devices [2, 25–27] but are lower than those of the InAs_*x*_P_1 − *x*_ alloy nanowire ones [13], as summarized in Table 1, thus confirming the potential of the Er-CdS NR for multicolor detection applications.

**Table 1:** A comparison of the critical parameters between this work and various nanostructure photodetectors

Photodetectors	Peak response wavelength (nm)	*R*_*λ*_ (A/W)	EQE (%)	Ref.
InAs	2900	1668	7.15 × 10^4^	[13]
InAs_0.8_P_0.2_	2300	4998	2.75 × 10^5^	
InAs_0.52_P_0.48_	1700	5417	3.96 × 10^5^	
ZnS NB	320	0.12	50	[2]
ZnSe NW	400	22	6810	[25]
Sb_2_Se_3_ NW	600	8.0	1650	[26]
Zn_2_GeO_4_ NW	250	38	18,970	[27]
In_2_Se_3_ NW: α-phase/κ-phase	500	160/0.84	39,620/210	[28]
	600	130/0.70	26,830/140	
Er-CdS NR	457.5	3.46 × 10^4^	93,800	This work
	620	8.14 × 10^3^	16,280	
	955	9.19 × 10^3^	11,930	

**Fig. 6:**
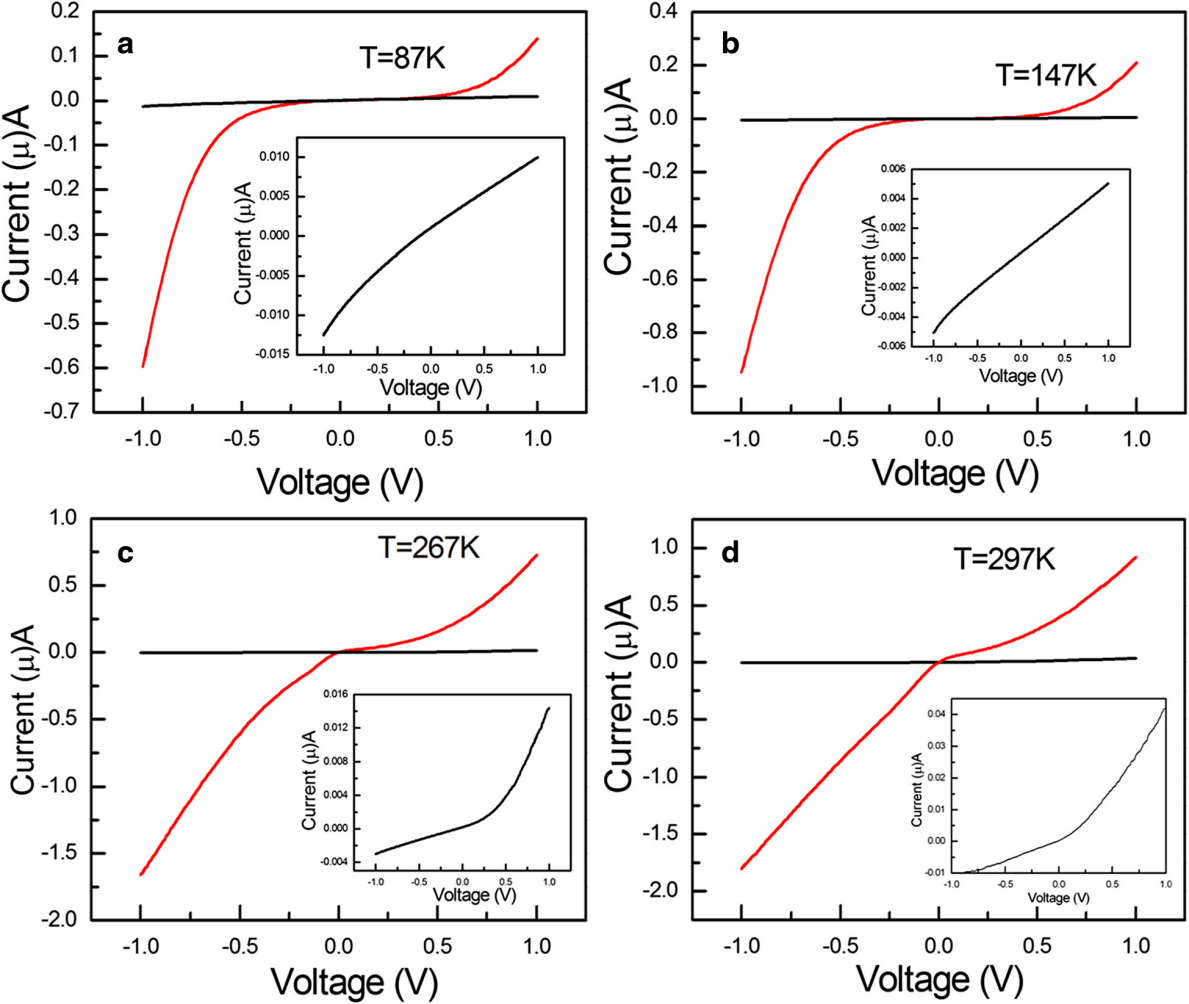
*I*-*V* curves of the Er-CdS NR detector under illumination with a lamp at different temperatures. **a** 87 K, **b** 147 K, **c** 267 K, and **d** 297 K

The PC evolutions of the Er-CdS NR on the temperature are shown in Fig. 6 under irradiation of a lamp. Interestingly, it is found that the PC current varies nonlinearly with applied voltages as the temperature changes from 87 to 297 K, which shown in Fig. 6. But the dark current behaves linearly with the applied voltages in the range of 87 to 147 K, as shown in the insets of Fig. 6a, b. In the meantime, it is also found that the photocurrent progressively has a linear shape in Fig. 6c, d, while on the opposition, the dark current has a curved shape.

**Fig. 7:**
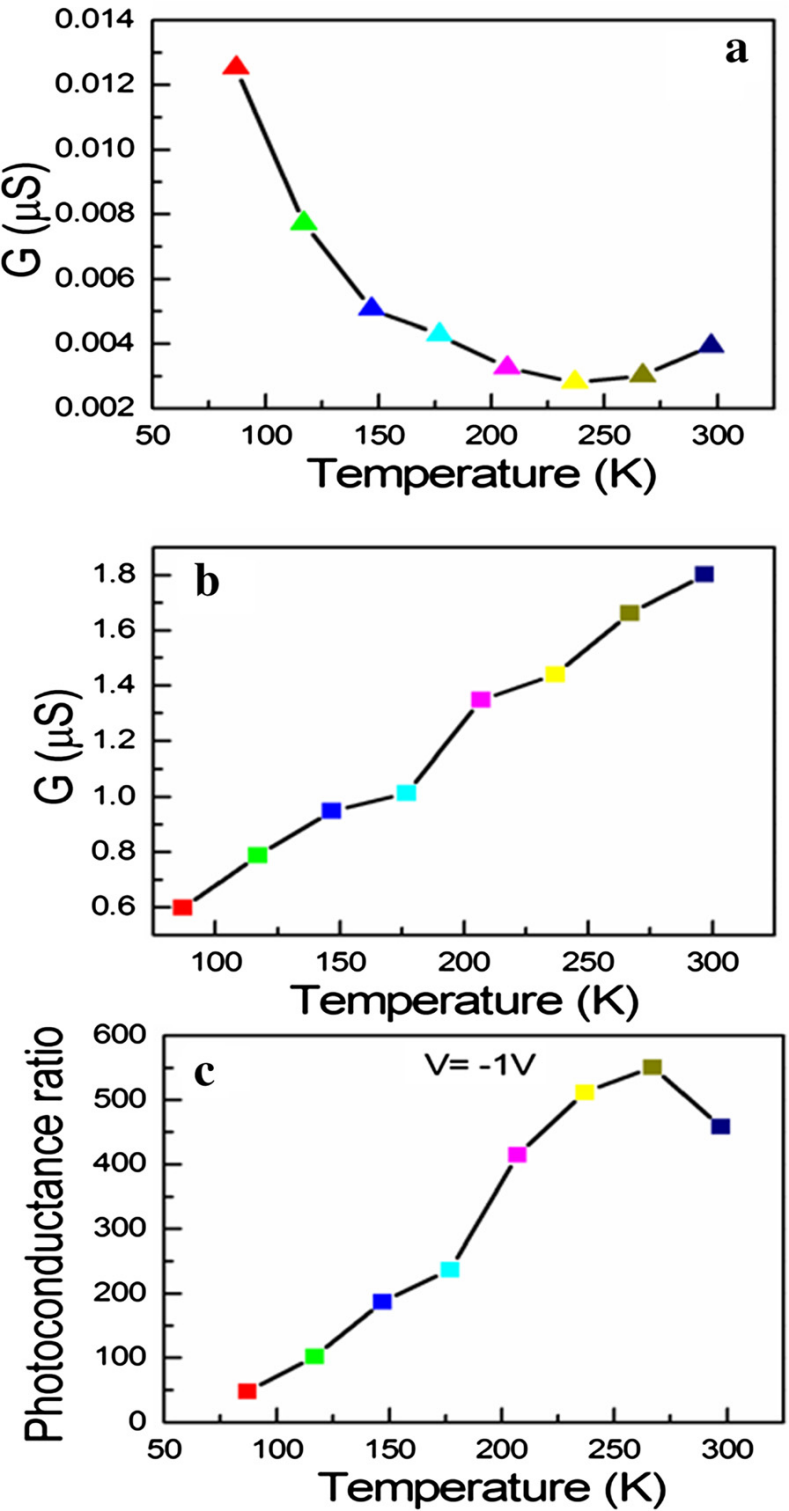
The relations between PC, DC, and temperature. **a** The dark conductance (DC) versus temperature curve of the Er-CdS NR detector. **b** Photoconductance (PC) dependence on the temperature of the Er-CdS NR detector. **c** The ratio of PC to DC versus temperature curve of the Er-CdS NR detector

To further investigate the surface-related processes and the transport processes of Er-CdS NRs, the dark conductance (hereafter denoted as DC), PC, and PC ratio were tested as a function of working temperature under illumination with an incandescent lamp and dark conditions in vacuum. Figure 7a exhibits the dependence of the dark conductance (*G*) on the temperature (*T*) at fixed intensity and voltage in vacuum (the pressure is in the range of 2.4~3.4 × 10^−1^ Torr). It is found that DC of the Er-CdS NR decreases with increasing operating temperature in the range of 87–237 K, indicating that the impurities are completely ionized and the intrinsic excitation is not primary [29], whereas the mobility decreases with increasing temperature; therefore, its DC reduces at the temperatures range of 87–237 K. However, DC of the Er-CdS NR increases with increasing temperature when *T* is larger than 237 K within the temperature range of 237–297 K, revealing that the intrinsic excitation quickly increases, and the yield of intrinsic carriers has more influence on DC of the Er-CdS NR than on the decrease of mobility. The intrinsic carriers have larger contributions to its DC. Therefore, DC decreases with increasing temperature, exhibiting the properties of intrinsic semiconductors. This result also reveals that the intrinsic carriers govern the dark conductance change. PC dependence of the Er-CdS NR on the temperature is shown in Fig. 7b under irradiation of incandescent light. It is found that PC of the CdS nanoribbon decreases with increasing temperature in the range of 87–297 K, which demonstrated that the intrinsic electron-hole (e-h) pairs are produced as soon as Er-CdS NR is illuminated by incandescent light and then the carriers are supplied drastically. As a result, its PC increases.

Figure 7c depicts the temperature-dependent conductance ratio under irradiation of incandescent light to that under dark conditions. It is seen that the PC ratio increases from 50 to 550 times when the temperature is from 86 to 267 K. But an abrupt decrease in the PC ratio is observed when temperature is raised above 267 K. Therefore, it is appropriate to detect incandescent light for the Er-CdS NR device at 267 K in vacuum.

To elucidate the adsorption effect, we also have measured the dark current and photocurrent of Er-CdS NR in lower vacuum. It is found that the dark current decreases, but the photocurrent ratios of the Er-CdS NR to the dark current are 350, 800, and 1500 under illuminations with torch light, weak table light, and strong table light, respectively (the pressure is 3 × 10^−3^ Torr), and the dark current (Additional file 1: Figure S1) has increased 2~6 times in vacuum (3 × 10^−3^ Torr) compared with that in air at room temperature. Combined with Fig. 3b, it is found that the surface absorption and desorption [30–32] (have important influence on) do affect not only the dark current but also the photocurrent ratio of the Er-CdS NR. The PC mechanism of Er-CdS NR is governed by the adsorption of oxygen as well as intrinsic carriers and ionization dopants.

## Conclusions

In conclusion, the photoconductance of the Er-CdS NR was investigated. The Er-CdS NR showed higher responses at 457.5, 620, and 955 nm. A multicolor photodetector of the Er-CdS NR was developed, which can simultaneously detect blue, red, and infrared light. The conductance of Er-CdS NRs in the dark decreases with increasing temperature in the range of 87–237 K, which indicates that the impurities are completely ionized and the intrinsic excitation is not primary. When *T* is larger than 237 K, the conductance of Er-CdS NRs in the dark increases with increasing temperature in the range of 237–297 K, and the intrinsic carriers have a larger contribution to the conductance. In addition, the PC ratio increases from 50 to 550 when the temperature is from 86 to 267 K, while the PC ratio decreases when the temperature is raised above 267 K, which is related to the excitation process of intrinsic carriers and impurities in the semiconductor.

## Additional file

Additional file 1:
**Supporting information.** Figure S1 *I*-*V* curves of the Er-CdS NR detector with pressure of 3 × 10^−3^ Torr. (a) Under the illumination of different light sources and (b) in the dark.
