# Supplementary figures and images for: Multicolor Photodetector of a Single Er3+-Doped CdS Nanoribbon
Source: Nanoscale Res Lett. 2015 Jul 8;10:285. doi: 10.1186/s11671-015-0975-3 (PMC4495098; doi:10.1186/s11671-015-0975-3)

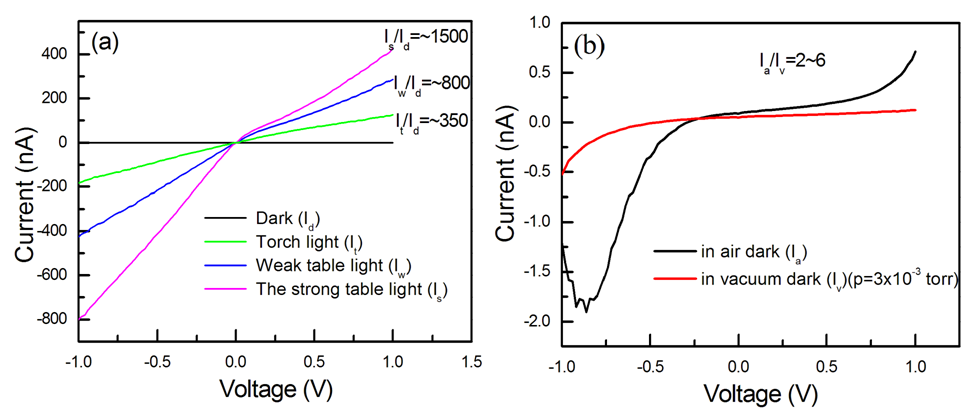

Supplement: Additional file 1: — Supporting information. Figure S1 I-V curves of the Er-CdS NR detector with pressure of 3 × 10^−3^ Torr. (a) Under the illumination of different light sources and (b) in the dark. [file 11671_2015_975_MOESM1_ESM.tiff]
